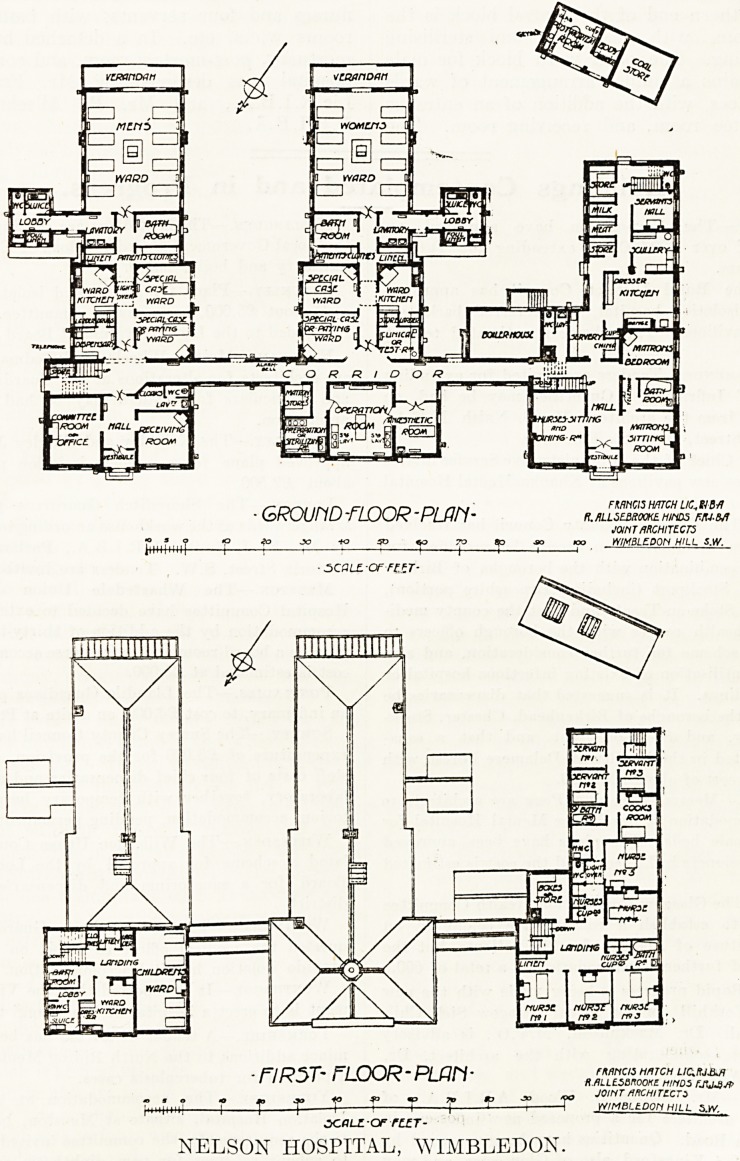# The New Nelson Hospital, Wimbledon

**Published:** 1912-09-21

**Authors:** 


### HOSPITAL ARCHITECTURE AND CONSTRUCTION.

**[Communications on this subject should be marked "Architecture" in the left-hand top corner of the envelope.]**

### The New Nelson Hospital, Wimbledon.

This hospital consists of three distinct blocks connected together by a corridor. Two of the blocks contain wards, the third being devoted to the kitchen offices and quarters for the resident staff. The centre block contains a ward for women for eight beds, with bath room, ward kitchen and sanitary offices, two single-bed wards, one of which is intended for paying patients, and a clinical or testing room. The long axis of the ward is roughly north-west by south-east, and at the southern end is an open verandah. The sanitary offices are placed in a projecting wing, with a cut-off lobby off the near or corridor end of the ward, thus leaving the whole of the ward free from projections which tend to

NELSON HOSPITAL, WIMBLEDON.

impede light and ventilation. The bath room also, instead of being placed in a projecting wing, is put, as it should be, close to the large ward, and is readily accessible to the single wards. There seems, however, to be no provision for a w.c. for nurses in either ward block—an omission which could easily have been remedied without undue extension of the sanitary wing.

At the northern end of the central block is the operation room, with anæsthetic and sterilising rooms adjoining. The other ward block for male patients contains a similar arrangement of wards and ward offices, with the addition of an entrance hall, committee room, and receiving room. The northern end of this block is carried up a storey higher, and contains a ward for eight children, ward kitchen, stores, w.c., and sink room.

The administrative block is two storeys in height and contains on the ground floor the matron's quarters, consisting of sitting room, bed room and bath room, nurses' sitting room, and the kitchen offices. On the floor above are bedrooms for five nurses and four servants, with bath rooms, box rooms w.c.s, etc. In a detached building are a mortuary, *post-mortem* room, and coal store. The hospital was designed by Mr. Francis Hatch, Lic.R.I.B.A., and Mr. R. Allsebrooke Hinds, F.R.I.B.A.

## Figures and Tables

**Figure f1:**